# Developing guidelines for reporting embedded recruitment trials

**DOI:** 10.1186/1745-6215-16-S2-O12

**Published:** 2015-11-16

**Authors:** Vichithranie W Madurasinghe, Sandra Eldridge, Gordon Forbes

**Affiliations:** 1 Queen Mary University of London, London, UK; 2 The University of Manchester, Manchester, UK

## The problem

Recruitment to clinical trials is problematic with many failing to recruit to target and within time. Embedding recruitment trials within effectiveness trials may provide a successful way to improve this. There are no guidelines for reporting such embedded trials. As part of the Systematic Techniques for Assisting Recruitment to Trials (START) project designed to test interventions to improve recruitment to trials, we developed guidelines for reporting embedded trials.

## The approach

We followed a three-phase guideline development process (1) pre-meeting literature review to generate items for the reporting guidelines (2) face-to-face consensus meetings to draft the reporting guidelines (3) post-meeting feedback review, and pilot testing, followed by finalisation of the reporting guidelines.

## Findings

We developed a reporting checklist based on the CONSORT statement 2010. Embedded trials evaluating recruitment interventions should follow the CONSORT statement 2010 and report all items listed as essential. We used a number of examples to illustrate key issues that arise in these trials and how best to report them, including how to deal with description of the host trial, the importance of describing items that may differ in the host and embedded trials such as the setting and the eligible population, and the importance of identifying clearly the point at which the recruitment interventions were embedded in the host trial.

## Consequence

Implementation of these guidelines will improve the quality of embedded recruitment trial reports while advancing the science, design and conduct of embedded trials as a whole.

